# Auricular Intradermal Acupuncture as a Supplementary Motor Rehabilitation Strategy in Poststroke Patients: A Randomized Preliminary Clinical Study

**DOI:** 10.1155/2020/5094914

**Published:** 2020-03-19

**Authors:** Dan Miao, Kuok-Tong Lei, Jing-Feng Jiang, Xin-Jun Wang, Hong Wang, Xiao-Ru Liu, Jia-Jia Zhang, Jia-Wei Xiong

**Affiliations:** ^1^Key Laboratory of Acupuncture and Medicine Research of Ministry of Education, Nanjing University of Chinese Medicine, Nanjing 210023, China; ^2^Affiliated Hospital of Nanjing University of Traditional Chinese Medicine, Nanjing 210023, China; ^3^The Second Affiliated Hospital of Nanjing University of Chinese Medicine, Nanjing 210017, China

## Abstract

We have explored the potential of auricular intradermal acupuncture (AIA) in standard rehabilitation and acupuncture treatment for motor recovery in poststroke patients. This was a randomized, controlled preliminary clinical study in which the patients were randomly assigned to the CT group (conventional treatment, standard rehabilitation, and routine acupuncture) or AIA group (AIA combined with conventional treatment) and underwent 6 sessions in 1 week (6 days). Standard procedures and previously reported acupuncture points were used. Clinical outcomes were measured by the Fugl-Meyer motor assessment (FMA) of flexor and extensor synergy movement (FSM and ESM) of the upper and lower extremities (UE and LE) at days 0, 3, and 6. The assessment was performed by blinded assessors. The AIA group showed a significant increase in FMA-UE/FMA-LE scores on day 3 (*P*=0.012 and 0.001, respectively) and day 6 (*P*=0.041 and *P* < 0.001, respectively), but this was not observed in the CT group. Furthermore, unlike the CT group, the AIA group exhibited a significant increase in the FMA-LE score on day 3 (*P*=0.004) and the FMA-UE scores on day 6 (*P*=0.048). Finally, the correlation between ESM and FMA-UE/FMA-LE was higher than that between FSM and FMA-UE/FMA-LE after treatment: for ESM and UE, *r* = 0.759, *P*=0.007; for ESM and LE, *r* = 0.697, *P*=0.003; for FSM and UE, *r* = 0.604, *P*=0.049; for FSM and LE, *r* = 0.347, *P*=0.188. AIA is useful for motor rehabilitation in poststroke patients, particularly in terms of improving extensor synergy. This trial is registered with CHiCTR1800020150.

## 1. Introduction

Stroke was one of the leading causes of death and disability-adjusted life years (DALYs) in China in 2017 [1]. Poststroke hemiplegia is one of the most severe complications, and it has an adverse effect on the quality of life of patients. In China, standard rehabilitation and traditional Chinese acupuncture are the routine treatment strategies. However, the potential value of acupuncture in terms of motor recovery is still controversial [2, 3]. Nonetheless, the benefits of craniofacial microacupuncture techniques, such as scalp acupuncture [4], eye acupuncture [5], and auricular acupuncture [6], have been demonstrated clinically in cases of hemiplegia. Although auricular acupuncture with penetrative needling methods has not been widely used in the treatment of stroke, further investigations are warranted.

The brainstem, as a supraspinal center, plays an important role in maintaining posture and voluntary movement. Recently, emerging evidence indicated vagus nerve stimulation as well contributed to the increase of Fugl-Meyer assessment (FMA) on the upper limb after stroke [7, 8]. Transcutaneous auricular branch vagus nerve stimulation (taVNS), a noninvasive alternative therapy, has similar benefits to invasive vagus nerve stimulation in terms of enhancing the effects of physiotherapy for upper limb motor recovery after stroke [7]. These findings indicate that poststroke motor rehabilitation could benefit from more direct neural pathway stimulation, particularly the stimulation of nuclei in the brainstem that are concerned with motor control. In this regard, the craniofacial pathway may prove to be effective as a target neural pathway.

For the clinical application of these rehabilitation methods, safety, feasibility, and affordability must be considered, in addition to efficacy. One method that meets all these criteria is the craniofacial microacupuncture system (including scalp acupuncture, auricular acupuncture, and eye acupuncture), which is a peripheral neurostimulation technique [9]. Auricular acupuncture has an exclusive feature because of the sophisticated nerve network in the organ. Any site in the external ear is well supplied through a unique surface distribution of the vagal branch, trigeminal nerves, and C2 to C3 branches of the cervical plexus [10]. Considering the role of taVNS in motor rehabilitation [7], acupuncture of the afferent nerve of the external ear may trigger the brainstem network to regulate the supraspinal neural pathways of motor control. Therefore, auricular intradermal acupuncture (AIA) may have value in motor rehabilitation after stroke as a supplementary method to conventional exercise training and acupuncture treatment. In order to explore this possibility, we conducted a randomized controlled clinical preliminary study of AIA combined with rehabilitation and routine acupuncture [11] for the treatment of motor dysfunction after stroke.

## 2. Methods

### 2.1. Study Design

We conducted a randomized, controlled, clinical preliminary study with blinded assessment on 42 patients with hemiplegia after stroke, who were being treated at the Affiliated Hospital of Nanjing University of Chinese Medicine. The study protocol was approved by the Chinese Clinical Trial Registry (approval no. 2017w1201) and registered at http://www.chictr.org.cn (registration no. CHiCTR1800020150).

### 2.2. Inclusion Criteria

Patients who met all of the following criteria were included: (1) diagnosis of cerebral infarction or cerebral hemorrhage according to the criteria of the Fourth National Cerebrovascular Disease Conference [12], confirmed by computed tomography or magnetic resonance imaging; (2) hemiplegia after stroke; (3) affected upper extremity and/or lower extremity classified as Brunnstrom stage III; (4) no more than 4 months after the episode of stroke; and (5) age between 30 and 80 years.

### 2.3. Exclusion Criteria

Patients who met any one of the following criteria were excluded: (1) serious disease of the heart, liver, kidney, or other organs; (2) somatic pain and limited mobility due to other diseases; (3) unconscious or presence of cognitive impairment; (4) presence of complications such as unilateral neglect and severe proprioception dysfunction; (5) blindness; and (6) subluxation of the upper extremities. It should be noted that patients with subluxation were not excluded, if the lower extremities were classified under Brunnstrom stage III.

### 2.4. Withdrawal from the Intervention

Patients who were unable to complete all the sessions of intervention were excluded from the study analysis. When patients withdrew, investigators contacted them or their families as soon as possible to record their reasons for withdrawal and the time of the last treatment. All the data for such cases have been carefully recorded and saved.

### 2.5. Randomization

Randomized allocation of the participants to the study groups was performed by an independent researcher. Based on computer-generated random numbers that were assigned to participants according to their order of enrollment, those with an even number were allocated to the AIA group and the others were allocated to the CT group. Both the therapist and the participants were aware of the treatment assigned. Only the evaluator was blinded to the assignment.

### 2.6. Study Interventions

#### 2.6.1. Rehabilitation

All participants underwent conventional poststroke rehabilitation exercise training. To develop voluntary movement independent of synergies, the Bobath, Brunnstrom, and Rood techniques were combined for exercise training. All tasks were guided by a physiotherapist. Rehabilitation training was performed during AIA for approximately 60 min per day, for a total of 6 sessions in 1 week.

#### 2.6.2. Routine Acupuncture

All participants underwent routine acupuncture. For routine acupuncture, disposable, sterilized, filiform needles that were 0.30 mm in diameter and 40 mm/75 mm in length (Hwato, Suzhou, China) were inserted into the skin at the following acupoints [5] on the affected side: LI15 (*Jianyu*), LI11(*Quchi*), LI10 (*Shousanli*), LI4 (*Hegu*), SJ5 (*Waiguan*), GB30 (*Huantiao*), GB34 (*Yanglingqua*n), ST36 (*Zusanli*), ST41 (*Jiexi*), and BL60 (*Kunlun*). The needles were retained for 30 min per day for a total of 6 sessions for 1 week.

#### 2.6.3. Auricular Intradermal Acupuncture

**Figure 1:**
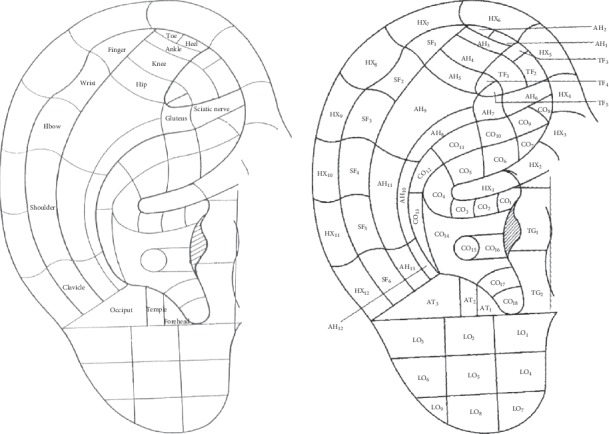
Schematic diagram of the standard location of auricular points and standard auricle partition code (according to the *Nomenclature and Location of Auricular Points [GB/T 13734-2008]*).

*(1) Auricular Acupoints*. According to the Standardization Administration of People's Republic of China's nomenclature and location of auricular points (GB/T 13734-2008) (Figure 1), the main acupoints on the affected side were AT3 (*occiput*)-AT2 (*temple*)-AT1 (*forehead*), located in the antitragus zone. The other acupoints corresponding to the upper or lower affected extremity were SF4, 5 (*shoulder*)-SF6 (*clavicle*), and SF3 (*elbow*)-SF2 (*wrist*)-SF1 (*finger*), located in the scaphe zone, and AH7 (gluteus)-AH6 (*sciatic nerve*), AH5 (*hip*)-AH4 (*knee*), and AH4 (*knee*)-AH3 (*ankle*)-AH2 (*toe*), located in the antihelix zone.

**Figure 2:**
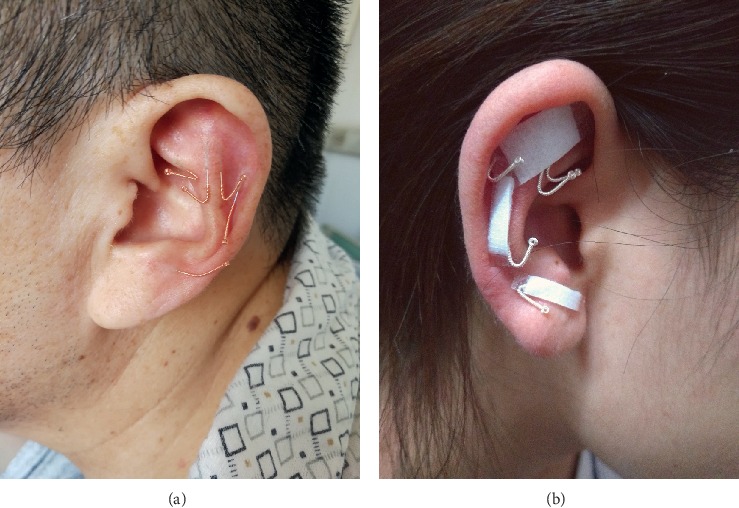
Images of the ear taken during the AIA procedure (a) with the needles not hidden and (b) with the needles hidden using a surgical tape.

*(2) Procedure*. Patients were requested to remain in the seated or supine position, and the skin of the auricle was disinfected with iodophors. Disposable, sterilized, filiform needles that were 0.18 mm in diameter and 10 mm in length (Emperor, Zhenjiang, China) were used from one point to another on a relatively flat surface within the regions above (i.e., from AT3 to AT1). Before acupuncture, the needle handles were bent so that they could be easily hidden and fixed. The horizontal acupuncture method (angle, <10°) was used to penetrate the skin of the auricle, without touching the auricular cartilage to avoid any pain. The intradermal area, rather than the subcutaneous region, was penetrated as much as possible. After acupuncture, the needles were fixed on the surface of the auricle using a medical tape. The needles were retained for 4 h in each session, and 6 sessions were conducted in a week for a total duration of 1 week (Images of the AIA procedure are shown in Figure 2).

### 2.7. Data Collection Methods

The first assessment was performed soon after the eligible participants provided their consent. Outcome measures were obtained from participants at baseline (day 0), day 3, and the end of the treatment (day 6). The information collected included patient history, Brunnstrom stage, and FMA score. An evaluator underwent the relevant training before conducting the assessment. In particular, their understanding of the scoring rules for written material was confirmed.

### 2.8. Outcome Assessment

#### 2.8.1. Fugl-Meyer Motor Assessment

The FMA scale assesses flexor synergy movement (FSM), extensor synergy movement (ESM), movement combining synergy, movement out of synergy, wrist, hand, and coordination/speed. It is divided into two parts, including an assessment of the upper extremities (FMA-UE: 33 items with a score range of 0–66) and the lower extremities (FMA-LE: 17 items with a score range of 0–34) [13]. Flexor synergy and extensor synergy movement are scored for the upper and lower extremities. The participants were evaluated before treatment (day 0), as well as on day 3 and after treatment (day 6).

#### 2.8.2. Statistical Analysis

SPSS 22.0 for Windows (SPSS Inc., Chicago, IL, USA) was used to analyze the data. The participants' demographic characteristics were presented as frequency and percentage, mean with SD, and median with interquartile range (IQR). For the continuous variables age, stroke duration, and FMA score, the Student's *t*-test (normal distribution) or nonparametric tests (nonnormal distribution) were used to compare the baseline values between the two groups. The categorical variables gender, stroke type, and paretic limbs were analyzed using the Fisher exact test. For observations at multiple time points, the FMA scores were analyzed using repeated measures analysis of variance (ANOVA). If the spherical hypothesis was proven according to Mauchly's spherical test, the sphericity correction factor was used. Otherwise, the Greenhouse–Geisser correction coefficient was used for correction. The significance level was set at 95% (two-sided *P* value, alpha <0.05). Finally, Spearman correlational analysis was used to investigate correlations between the increase in FSM/ESM and FMA-UE/FMA-LE after treatment.

## 3. Results

**Figure 3:**
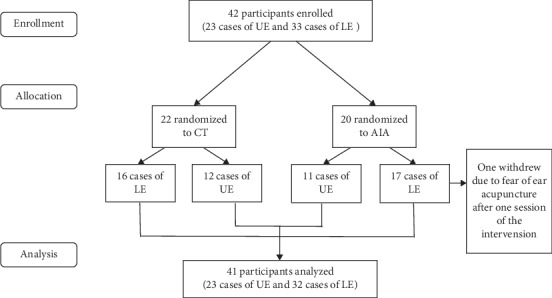
Flow chart showing the patient selection process. *AIA, auricular intradermal acupuncture; CT, conventional treatment; UE, upper extremity; LE, lower extremity.*

Of 356 patients who were initially screened, 42 patients who met the eligibility criteria were recruited. Finally, 41 patients (19 in the AIA group and 22 in the CT group, median age = 67 years, IQR = 54–74 years) completed the treatment, and a total of 23 upper extremities and 32 lower extremities were assessed. One patient in the AIA group withdrew due to fear of needling pain. The baseline characteristics (Table 1), including age, gender, paretic limbs, duration of disease, and the baseline scores of FMA-UE and FMA-LE (Table 2), were comparable between the AIA group and the CT group. A flow chart of the selection process is shown in Figure 3.

In terms of safety, 7 patients who underwent AIA experienced slight bleeding after needle penetration. No other adverse events related to acupuncture occurred.

**Figure 4:**
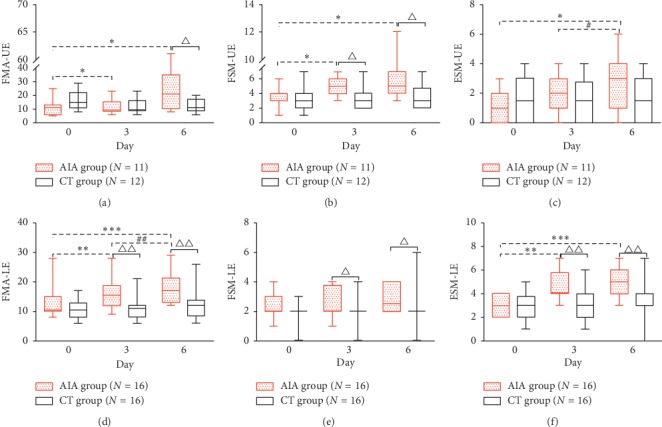
Fugl-Meyer motor assessment of the control and treatment groups. a-f: FMA-UE, FSM-UE, ESM-UE, FMA-LE, FSM-LE, and ESM-LE scores on days 0, 3, and 6 for the two groups. The FMA scores are expressed as median (IQR). *FMA-UE, Fugl-Meyer assessment of the upper extremity; FSM-UE, flexor synergy movement of the upper extremity; ESM-UE, extensor synergy movement of the upper extremity; FMA-LE, Fugl-Meyer assessment of the lower extremity; FSM-LE, flexor synergy movement of the lower extremity; ESM-LE, extensor synergy movement of the lower extremity.*

As shown in Figure 4, the AIA group had higher FMA-LE values on day 3 (*P*=0.004) and higher FMA-UE and FMA-LE values on day 6 (*P*=0.048 and 0.001, respectively) than the CT group (Figures 4(a) and 4(d)). Furthermore, FMA subitem analysis of FSM/ESM showed that there was a significant difference in the median score change for FSM-UE, FSM-LE, and ESM-LE between the two groups (*P* < 0.05 for all) on day 6 (Figures 4(b), 4(e), and 4(f)), but the difference was not significant for ESM-UE (*P*=0.168) (Figure 4(c)). Furthermore, patients in the AIA group exhibited a significant increase in the FMA-UE and FMA-LE scores on day 3 (*P*=0.012 and 0.001, respectively) and day 6 (*P*=0.041 and <0.001, respectively), but this was not observed in the CT group (*P* > 0.05) (Figures 4(a) and 4(d)). A 6.0- and 4.0-point change in the FMA-UE and FMA-LE scores, respectively, was found in the AIA group after the 6-day treatment, but the corresponding change in the CT group was 0 points and 0.5 points for FMA-UE and FMA-LE respectively.

As shown in Tables 2 and 3, correlation analysis in the AIA group indicated that the correlation between the increase in ESM and the increase in the FMA-UE/FMA-LE scores was higher than that between FSM increase and FMA-UE/FMA-LE increase after treatment, as the following displayed: ESM-UE (*r* = 0.759, *P*=0.007), ESM-LE (*r* = 0.697, *P*=0.003), FSM-UE (*r* = 0.604, *P*=0.049), and FSM-LE (*r* = 0.347, *P*=0.188).

## 4. Discussion

The present study explores the application of AIA along with standard rehabilitation and acupuncture therapy in stroke patients at Brunnstrom stage III. The preliminary data look promising, as the findings indicate that AIA improved flexor muscle synergy in the upper and lower extremities and extensor muscle synergy in the lower limbs. The changes observed with AIA were consistent with the features of reticular formation.

Auricular acupuncture with penetrative needling methods has been reported to promote limb myodynamia and neurofunction in patients with acute cerebral infarction [6]. As the nerves of the outer ear are in the dermis and in the perichondrium [14], AIA and auricular needle penetration may have similar efficacy. For the AIA procedure, it is important that the needles penetrate the dermal lamina rather than the subdermal lamina. This is to ensure that the perichondrium remains untouched and the subjects do not experience any pain. Therefore, the AIA procedure is almost imperceptible and painless. Additionally, with the way of AIA stimulation transformed dot stimulus of conventional auricular acupuncture to line stimulus, the spatial summation augmented via the region stimulated enlarged.

Recently, experiments and clinical studies have demonstrated that the VNS has a regulatory effect on muscle strength [15–17] and proximal dystonia [18, 19] in hemiplegic patients. The reported findings indicate that the vagus nerve may be involved in circuits related to motor control, in addition to visceral organ innervation. This implies that cranial nerve stimulation will be more efficient in motor rehabilitation.

Previous studies on VNS/taVNS stimulation have only reported improvement of the upper limb in poststroke patients, and they have not analyzed the effects on flexor or extensor synergy [7, 8]. Our study makes an important contribution in this regard, as our results demonstrate improvement in both flexor and extensor synergy with AIA. Additionally, the effects of the AIA protocol employed in this study were observed within a short time period of 6 days. Previous studies have reported much longer treatment periods of 90 days and 6 weeks [8, 20]. Thus, the protocol used here had a faster treatment effect.

The acupoints used in routine body acupuncture have been reported to have no value in standard rehabilitation strategies [11]. However, standard acupuncture protocols are still widely used in China for stroke rehabilitation as the limitation of the study, such as lack of subunit analysis. Therefore, the result of our study would not be biased by the acupoint protocol. On all account, the acupoints used in the present study cannot be efficient in 6 days because it was testified no value in 30 days.

## 5. Conclusion

The findings of the present study indicate that AIA adds considerable value to standard motor rehabilitation and routine acupuncture therapy in Brunnstrom stage III patients undergoing rehabilitation after stroke. In particular, AIA was found to improve extensor synergistic movement and acceleration in patients undergoing rehabilitation.

## Figures and Tables

**Table 1:** Baseline characteristics of the patients.

Variables	Upper extremity			Lower extremity		
	AIA (*n* = 11)	CT (*n* = 12)	*P*	AIA (*n* = 16)	CT (*n* = 16)	*P*
Age (y), mean (SD)^a^	62.23 (13.44)	65.22 (11.14)	0.420	59.25 (16.14)	70.60 (8.55)	0.060
Gender, *n* (%)^b^						
Male	10 (90.9)	7 (58.3)		13 (81.3)	11 (68.8)	
Female	1 (9.1)	5 (41.7)	0.155	3 (18.8)	5 (31.3)	0.685
Stroke duration (d), median (IQR)^c^	15.0 (5.0–23.0)	17.5 (12.0–27.3)	0.196	18.5 (7.3–57.0)	11.0 (4.3–31.0)	0.157
Stroke type, *n* (%)^b^						
Ischemia	10 (90.9)	10 (83.3)		10 (62.5)	12 (75.0)	
Hemorrhage	1 (9.1)	2 (16.7)	1.000	6 (37.5)	4 (25.0)	0.704
Paretic limb, *n* (%)^b^						
Right	6 (54.5)	4 (33.3)	0.414	8 (50.0)	4 (25.0)	
Left	5 (45.5)	8 (66.7)		8 (50.0)	12 (75.0)	0.273

Data are expressed as numbers, mean ± standard deviation, or median (IQR). ^a^Student's *t*-test, ^b^Fisher exact test, and ^c^Wilcoxon rank-sum test.

**Table 2:** Correlation between the changes in FSM-UE/ESM-UE and FMA-UE by Spearman correlation analysis.

Group	Day	Variable	Changes in FMA-UE scores	
			Correlation coefficient (*r*)	*P*
AIA (*n* = 11)	3	Changes in FSM-UE scores	0.645	0.032
		Changes in ESM-UE scores	0.691	0.019
	6	Changes in FSM-UE scores	0.604	0.049
		Changes in ESM-UE scores	0.759	0.007

CT (*n* = 12)	3	Changes in FSM-UE scores	0.721	0.008
		Changes in ESM-UE scores	0.573	0.051
	6	Changes in FSM-UE scores	0.763	0.004
		Changes in ESM-UE scores	0.235	0.462

**Table 3:** Correlation between the changes in FSM-LE/ESM-LE and FMA-LE by Spearman correlation analysis.

Group	Day	Variable	Changes in FMA-LE scores	
			Correlation coefficient (*r*)	*P*
AIA (*n* = 16)	3	Changes in FSM-LE scores	0.145	0.591
		Changes in ESM-LE scores	0.755	0.001
	6	Changes in FSM-LE scores	0.347	0.188
		Changes in ESM-LE scores	0.697	0.003

CT (*n* = 16)	3	Changes in FSM-LE scores	0.664	0.005
		Changes in ESM-LE scores	0.748	0.001
	6	Changes in FSM-LE scores	0.493	0.052
		Changes in ESM-LE scores	0.874	<0.001

## Data Availability

The data that support the findings of this study are openly available in Clinical Trial Management Public Platform at http://wwww.medresman.org:22280/login.aspx (reg no. CHiCTR1800020150).

## Abbreviations

AIA: Auricular intradermal acupuncture
CT: Conventional treatment
VNS: Implanted vagus nerve stimulation
taVNS: Transcutaneous auricular branch vagus nerve stimulation
FMA: Fugl-Meyer motor assessment
FMA-UE: Fugl-Meyer assessment of the upper extremity
FSM-UE: Flexor synergy movement of the upper extremity
ESM-UE: Extensor synergy movement of the upper extremity
FMA-LE: Fugl-Meyer assessment of the lower extremity
FSM-LE: Flexor synergy movement of the lower extremity
ESM-LE: Extensor synergy movement of the lower extremity.

## References

[1] Zhou M., Wang H., Zeng X. (2019). Mortality, morbidity, and risk factors in China and its provinces, 1990–2017: a systematic analysis for the global burden of disease study 2017. *The Lancet*.

[2] Sze F. K.-H., Wong E., Or K. K. H., Lau J., Woo J. (2002). Does acupuncture improve motor recovery after stroke?. *Stroke*.

[3] Wu P., Mills E., Moher D., Seely D. (2010). Acupuncture in poststroke rehabilitation: a systematic review and meta-analysis of randomized trials. *Stroke*.

[4] Qi L., Han Z., Zhou Y. (2018). Dynamic scalp acupuncture combined with PNF therapy for upper limb motor impairment in ischemic stroke spastic hemiplegia. *Zhong Guo Zhen Jiu*.

[5] Bai Z. H., Zhang Z. X., Li C. R. (2015). Eye acupuncture treatment for stroke: a systematic review and meta-analysis. *Evidence-Based Complementary and Alternative Medicine*.

[6] Li C. F., Jia C. S., Li X. F. (2010). Effect of penetrative needling of otopoints combined with body acupuncture on limb myodynamia and neurofunction in patients with acute cerebral infarction. *Zhen Ci Yan Jiu*.

[7] Redgrave J. N., Moore L., Oyekunle T. (2018). Transcutaneous auricular vagus nerve stimulation with concurrent upper limb repetitive task practice for poststroke motor recovery: a pilot study. *Journal of Stroke and Cerebrovascular Diseases*.

[8] Kimberley T. J., Pierce D., Prudente C. N. (2018). Vagus nerve stimulation paired with upper limb rehabilitation after chronic stroke. *Stroke*.

[9] Kagitani F., Uchida S., Hotta H. (2010). Afferent nerve fibers and acupuncture. *Autonomic Neuroscience*.

[10] Peuker E. T., Filler T. J. (2002). The nerve supply of the human auricle. *Clinical Anatomy*.

[11] Sze F. K.-H., Wong E., Yi X., Woo J. (2002). Does acupuncture have additional value to standard poststroke motor rehabilitation?. *Stroke*.

[12] The Fourth Academic Seminar of the Chinese Society for Neuroscience (1996). Major diagnostic points of cerebrovascular disease. *Chinese Journal of Neurology*.

[13] Sullivan K. J., Tilson J. K., Cen S. Y. (2011). Fugl-meyer assessment of sensorimotor function after stroke. *Stroke*.

[14] Bermejo P., Lopez M., Larraya I. (2017). Innervation of the human cavum conchae and auditory canal: anatomical basis for transcutaneous auricular nerve stimulation. *BioMed Research International*.

[15] Khodaparast N., Hays S. A., Sloan A. M. (2013). Vagus nerve stimulation during rehabilitative training improves forelimb strength following ischemic stroke. *Neurobiology of Disease*.

[16] Hays S. A., Ruiz A., Bethea T. (2016). Vagus nerve stimulation during rehabilitative training enhances recovery of forelimb function after ischemic stroke in aged rats. *Neurobiology of Aging*.

[17] Pruitt D. T., Schmid A. N., Kim L. J. (2016). Vagus nerve stimulation delivered with motor training enhances recovery of function after traumatic brain injury. *Journal of Neurotrauma*.

[18] Kampusch S., Kaniusas E., Szeles J. C. (2013). Expected effects of auricular vagus nerve stimulation in dystonia. *Biomedical Engineering/Biomedizinische Technik*.

[19] Kampusch S., Kaniusas E., Széles J. C. (2015). Modulation of muscle tone and sympathovagal balance in cervical dystonia using percutaneous stimulation of the auricular vagus nerve. *Artificial Organs*.

[20] Dawson J., Pierce D., Dixit A. (2016). Safety, feasibility, and efficacy of vagus nerve stimulation paired with upper-limb rehabilitation after ischemic stroke. *Stroke*.

